# Remote monitoring and digital triage in patients awaiting TAVI: from feasibility to implementation

**DOI:** 10.1093/ehjdh/ztaf128

**Published:** 2025-11-03

**Authors:** Ioannis Skalidis, Mario Togni, Stephane Cook

**Affiliations:** Department of Cardiology, University & Hospital Fribourg, Fribourg 1708, Switzerland; Department of Cardiology, University & Hospital Fribourg, Fribourg 1708, Switzerland; Department of Cardiology, University & Hospital Fribourg, Fribourg 1708, Switzerland

The COVID-19 era and its aftermath have accelerated the adoption of remote patient monitoring (RPM) and digital health pathways across cardiovascular care. In structural heart disease, where waiting times and procedural backlogs remain critical determinants of outcome, these technologies promise to transform traditional models of patient follow-up and prioritization. Yet, empirical data supporting their safety, feasibility, and impact on hard endpoints have been scarce, particularly among elderly, high-risk populations awaiting transcatheter aortic valve implantation (TAVI). Against this backdrop, recent evidence from Imperial College Healthcare NHS Trust provides an important step towards integrating digital triage into the real-world continuum of valvular care.

The study by Kelshiker *et al*. offers timely and methodologically robust evidence on the feasibility and safety of an RPM-based prioritization pathway for patients awaiting TAVI.^1^ In an era of constrained procedural capacity and rising waiting-list morbidity, their prospective, propensity-matched cohort provides an important proof-of-concept that digital health technologies may mitigate the clinical impact of procedural delays. The authors are to be commended for addressing a pressing systems-level problem with a pragmatic, data-driven approach embedded within the NHS infrastructure.

The finding that RPM maintained safety despite significantly longer waiting times and accurately identified patients at risk of deterioration are clinically relevant. However, several scientific and operational aspects merit further discussion to optimize such pathways and to guide future multicentre implementation.

First, the relationship between monitoring sensitivity and clinical actionability deserves deeper exploration. The study demonstrated 100% sensitivity for predicting waiting-list death at the first escalation level but with very low positive predictive value (6%), indicating a substantial false-positive burden. While prioritizing sensitivity is ethically appropriate in high-risk cohorts, sustained high alert volumes can erode clinician responsiveness and inflate staffing requirements. Could algorithmic thresholds be optimized to balance safety with operational sustainability? The trade-off between sensitivity and specificity could be quantified using receiver-operating characteristics for varying escalation thresholds, potentially allowing calibration of the rule-based algorithm to achieve an optimal equilibrium. Integration of additional longitudinal variables (such as biometric signals from wearable sensors or objective functional parameters) may also enhance specificity while preserving sensitivity.

Second, the study highlights the critical role of system capacity in translating digital triage into tangible outcome benefits. Despite accurate risk identification, the median waiting time after escalation to an expedited TAVI date remained 35 days, exceeding national targets. Two of five patients admitted emergently following escalation died, underscoring that timely procedural access remains the rate-limiting step in preventing deterioration. Could RPM-derived alerts be integrated into real-time scheduling algorithms, linking escalation severity directly to procedural allocation priorities? Future studies might simulate alternative queue-management models to estimate potential reductions in morbidity and mortality under varying capacity scenarios.

Third, the implications for digital inclusion in elderly TAVI candidates warrant continued attention. Notably, 61% of participants engaged independently with the online platform and a further 6% with caregiver assistance, which remains remarkably high for a cohort with median age above 80 years. Nevertheless, one-third required telephone-based monitoring, indicating persistent structural and cognitive barriers to digital participation. Evaluating the comparative accuracy, adherence, and patient-reported experience between digital and telephone modalities could clarify whether analogue approaches achieve equivalent safety. Moreover, stratifying engagement by cognitive status, frailty, or socioeconomic indicators might help define which subgroups benefit most from graded digital support vs. alternative community-based strategies. How might future iterations of such programmes ensure equitable participation without compromising the quality of monitoring or clinical outcomes?

Beyond these key considerations, the study’s methodological transparency and particularly the use of a regional electronic health record for outcome ascertainment and rigorous propensity matching, strengthens its external validity. Yet, as the authors acknowledge, residual confounding remains possible in a single-centre observational design with a low event rate. Future research could adopt a stepped-wedge or cluster-randomized framework across multiple TAVI centres to quantify causal effects on mortality, hospitalization, and cost-effectiveness. Embedding formal implementation-science metrics (such as fidelity, adaptability, and workload impact) would also yield valuable insights into scalability.

Ultimately, this study advances the concept of ‘virtual queue management’ for high-risk cardiovascular interventions. By bridging traditional heart-team decision-making with continuous remote surveillance, it establishes an ethical and technological foundation for precision triage within resource-limited health systems. As waiting times for TAVI continue to expand globally, the next challenge is to transform RPM-derived data into actionable scheduling intelligence that equitably accelerates care for those most in need, without widening digital disparities.
